# Targeting the tumor stroma with an oncolytic adenovirus secreting a fibroblast activation protein-targeted bispecific T-cell engager

**DOI:** 10.1186/s40425-019-0505-4

**Published:** 2019-01-25

**Authors:** Jana de Sostoa, Carlos Alberto Fajardo, Rafael Moreno, Maria D. Ramos, Martí Farrera-Sal, Ramon Alemany

**Affiliations:** 1 ProCure Program, IDIBELL-Institut Català d’Oncologia, l’Hospitalet de Llobregat, El Prat de Llobregat, Spain; 2 VCN Biosciences S.L., Grifols Corporate Offices, Sant Cugat del Vallès, Spain

**Keywords:** Oncolytic adenovirus, Bispecific T-cell engager, Fibroblast activation protein, Tumor-associated stroma

## Abstract

**Background:**

Oncolytic virus (OV)-based therapies have an emerging role in the treatment of solid tumors, involving both direct cell lysis and immunogenic cell death. Nonetheless, tumor-associated stroma limits the efficacy of oncolytic viruses by forming a barrier that blocks efficient viral penetration and spread. The stroma also plays a critical role in progression, immunosuppression and invasiveness of cancer. Fibroblast activation protein-α (FAP) is highly overexpressed in cancer-associated fibroblasts (CAFs), the main cellular component of tumor stroma, and in this study we assessed whether arming oncolytic adenovirus (OAd) with a FAP-targeting Bispecific T-cell Engager (FBiTE) could retarget infiltrated lymphocytes towards CAFs, enhancing viral spread and T cell-mediated cytotoxicity against the tumor stroma to improve therapeutic activity.

**Methods:**

The bispecific T-cell Engager against FAP was constructed using an anti-human CD3 single-chain variable fragment (scFv) linked to an anti-murine and human FAP scFv. This FBiTE was inserted in the oncolytic adenovirus ICOVIR15K under the control of the major late promoter, generating the ICO15K-FBiTE. ICO15K-FBiTE replication and potency were assessed in HT1080 and A549 tumor cell lines. The expression of the FBiTE and the activation and proliferation of T cells that induced along with the T cell-mediated cytotoxicity of CAFs were evaluated by flow cytometry *in vitro.* *In vivo*, T-cell biodistribution and antitumor efficacy studies were conducted in NOD/*scid*/*IL2rg*^*−*^*/*^*−*^ (NSG) mice.

**Results:**

FBiTE expression did not decrease the infectivity and replication potency of the armed virus*.* FBiTE-mediated binding of CD3^+^ effector T cells and FAP^+^ target cells led to T-cell activation, proliferation, and cytotoxicity of FAP-positive cells *in vitro*. *In vivo,* FBiTE expression increased intratumoral accumulation of T cells and decreased the level of FAP, a marker of CAFs, in tumors. The antitumor activity of the FBiTE-armed adenovirus was superior to the parental virus.

**Conclusions:**

Combination of viral oncolysis of cancer cells and FBiTE-mediated cytotoxicity of FAP-expressing CAFs might be an effective strategy to overcome a key limitation of oncolytic virotherapy, encouraging its further clinical development.

**Electronic supplementary material:**

The online version of this article (10.1186/s40425-019-0505-4) contains supplementary material, which is available to authorized users.

## Background

Oncolytic viruses (OVs) are emerging as promising anti-tumor agents in cancer treatment, offering an attractive combination of tumor-specific cell lysis and intratumoral immune stimulation. Engineered OVs have been tested in several Phase I-III clinical trials, and Talimogene laherparepvec (Imlygic®), an Herpes Simplex Virus (HSV) expressing the granulocyte macrophage colony stimulating factor (GM-CSF), has been recently approved by FDA and EMA for the treatment of melanoma. Despite their potential, OVs have several limitations that should be tackle to improve their efficacy.

One of the major obstacles to successful oncolytic therapy is the presence of stroma in tumors, formed by different types of cells and extracellular matrix (ECM) compounds. Stroma not only creates physical barriers that limit oncolytic adenovirus (OAd) spread across the tumor, but also induces tumor progression by enhancing the survival, proliferation, stemness, metastasis, and an immunosuppressive microenvironment that limits tumor immunity, ultimately promoting cancer progression, but also enhancing resistance to therapy [1]. One attractive stromal target is the fibroblast activation protein-α (FAP), a transmembrane serine protease that is highly expressed on the cell surface of cancer-associated fibroblasts (CAFs), which represent the key component in the tumor microenvironment of many cancers [2]. Accordingly, several immunotherapeutic strategies to deplete FAP-expressing stromal cells have already been explored [3–11].

Another important hurdle for the efficacy of OVs is the host immune response to the OV. Antiviral immune responses can intrinsically limit OV infection, spread, and overall therapeutic efficacy. However, there is increasing evidence that virus-mediated destruction or damage of tumors can lead to an antitumor immune response [12]. Thus, novel strategies to minimize the antiviral immune response for successful virus growth and retreatment, but to stimulate antitumor responses, would provide an opportunity to tilt this balance in favor of the therapeutic benefit.

Based on the pro-tumorigenic functions of tumor stroma and the strong antiviral immune responses that limit OV therapy, the destruction of CAFs by arming OVs with FAP-targeting Bispecific T-cell Engagers (BiTEs) may mitigate the key limitations of OVs [3]. BiTE antibody constructs comprise tandemly-arranged single-chain variable fragments (scFvs). One scFv binds the TCR CD3ε subunit and the other binds a tumor-associated surface antigen (TAA). The simultaneous binding of the BiTE to the CD3 on T cells and to the TAA on target cells leads to the formation of the immunological synapse due to the close proximity of both membranes, leading to polyclonal T-cell activation, expansion and lysis of the protein-expressing target cells. Blinatumomab (Blincyto®), a first-in-class BiTE, has shown promise results for treating relapsed/refractory precursor B cell acute lymphoid leukemia (ALL) [13]. We have previously generated an anti-EGFR BiTE-armed OAd, which showed to improve T cell-mediated killing of cancer cells both *in vitro* and *in vivo* [14]*.*

Here we report the development of the OAd ICO15K-FBiTE encoding FAP-targeting BiTE to retarget infiltrated lymphocytes against FAP-expressing CAFs. We show the ability of ICO15K-FBiTE to induce strong and specific T-cell activation and proliferation upon infection, leading to T cell-mediated cytotoxicity of CAFs *in vitro* and enhanced antitumor activity due to FAP depletion *in vivo.*

## Methods

### Cell lines

Human cell lines A549 (lung adenocarcinoma), HEK293 (embryonic kidney), HT1080 (fibrosarcoma), A431 (vulval epidermoid carcinoma), Jurkat (T-cell leukemia) and HPAC (pancreatic adenocarcinoma) were obtained from the American Type Culture Collection (ATCC). Human CAFs pf179 (named as hCAFs) were kindly provided by Varda Rotter (Weizmann Insitute of Science, Israel). 293, 293mFAP and 293hFAP cell lines were obtained from Dr. Eric Tran (National Institutes of Health, Bethesda, MD). Murine CAFs were isolated from HPAC tumors as described [15]. To generate FAP-expressing cell lines, HT1080 and A431 cells were transduced with a lentivirus encoding either the mouse or the human FAP cDNA (Dharmacon). FAP-expressing cells were sorted and expanded. HT1080 cells stably expressing mouse FAP or human FAP are designated as HT-mFAP and HT-hFAP, respectively. A431 cells are designated as A431-mFAP and A431-hFAP. All tumor cell lines were maintained in Dulbecco’s modified Eagle’s medium (DMEM) supplemented with inactivated 10% fetal bovine serum (FBS, Invitrogen Carlsbad) and 1X Penicillin/Streptomycin (PS, Gibco) at 37 °C, 5% CO_2_ incubator, except for Jurkat cells which were maintained in RPMI-1640 medium. All cell lines were routinely tested for mycoplasma.

### Preparation of peripheral blood mononuclear cells and T cell isolation

All experiments were approved by the ethics committees of the University Hospital of Bellvitge and the Blood and Tissue Bank (BST) from Catalonia. Blood samples were obtained from the BST from Catalonia. Peripheral blood mononuclear cells (PBMCs) were isolated by ficoll density gradient centrifugation. PBMCs were treated with ACK lysis buffer (Lonza) and resuspended in RPMI-1640 medium supplemented with 10% FBS. T cells were isolated using the Rosette-Sep Human T Cell Enrichment Cocktail (STEMCELL Technologies). For stimulation, T cells were cultured with CD3/CD28-activating Dynabeads (Thermo Fisher Scientific) at 1:3 bead-to-cell ratio. For bioimaging studies, T cells were transduced with a lentivirus expressing GFP and the click beetle green luciferase (CBG) (multiplicity of infection (MOI) of 7) 24 h hours after activation. Cells were counted and fed every day until day 10, time point at which they were either used for functional assays or cryopreserved.

### FBiTE and construction of recombinant adenoviruses

FBiTE was generated by joining the scFvs anti-FAP and anti-CD3ɛ with a GGGGS flexible linker. The anti-CD3 scFv sequence of the Blinatumomab BiTE was obtained from patent application WO2004106381. The anti-FAP sequence (FAP5) was derived from patent application US 2009/0304718 A1 and showed affinities of 5 nM for human FAP and 0.6 nM for mouse FAP [10]. The FAP5 and anti-CD3 variable regions were connected by a (G_4_S_1_)_3_ and a (G_2_S_1_)_4_GG linker, respectively. The FBiTE was arranged V_L_(FAP5)-V_H_(FAP5)-V_H_(CD3)-V_L_(CD3) and contained an N-terminal signal peptide derived from the mouse immunoglobulin light chain for mammalian secretion, and a FLAG tag at the C-terminal for detection. The FBiTE construct was optimized for human codon usage and synthesized by Baseclear (pUC57-FBiTE plasmid, Baseclear). The genome of ICO15K-FBiTE was obtained by recombineering in bacteria as described [16]. HEK293 cells were transfected with the resulting plasmid pAdZ-ICO15K-FBiTE with calcium phosphate standard protocol. ICO15K-FBiTE was plaque-purified and further amplified in A549 cells. Viruses were double purified by cesium chloride gradient centrifugation and tittered using anti-hexon staining.

### Production of FBiTE-containing supernatants

A549 cells (1 × 10^7^) were infected at MOI of 20 with ICO15K or ICO15K-FBiTE. 72 h post-infection, supernatants were collected and centrifuged 5 min at 1200 g to eliminate detached cells. Supernatants from uninfected cells were used as a mock control. For binding assays, supernatants were concentrated (approximately 20x) with Amicon Ultra-15 filter units with a molecular weight cutoff of 30 kDa (Merck Millipore). Aliquots of the supernatant were stored at − 20 °C for future analysis.

### Antibodies and flow cytometry

Flow cytometry analysis was performed on a Gallios cytometer (Beckman Coulter) and data was processed with FlowJo v7.6.5 (Tree Star). Murine FAP expression was detected with the mouse 73.3 antibody kindly provided by Dr. Ellen Puré (Abramson Family Cancer Research Institute, Philadelphia, Pennsylvania) and human FAP expression was detected with F19 hybridoma (ATCC). For analysis of T cell populations, antibodies CD3 (clone OKT3), CD4 (OKT4) and CD8 (SK1) (Biolegend) were used. The FITC-conjugated anti-FLAG M2 monoclonal antibody (Sigma Aldrich) was used to detect the BiTE in binding assays. In each case, appropriated isotype controls were used (Santa Cruz Biotechnology).

### Binding assays

Binding assays were performed with HT1080 cells transfected with either human or murine FAP antigen or CD3^+^ Jurkat cells. HT1080 (2 × 10^5^) or Jurkat (1 × 10^5^) cells were incubated on ice for one hour with the concentrated or unconcentrated supernatants. BiTE binding was determined by flow cytometry using anti-FLAG M2-FITC antibody (Sigma Aldrich).

### In vitro co-culture experiments

Tumor cells (3 × 10^5^) and PBMCs or T cells (effector-to-target ratio of 5) were seeded in 96-well plates in 100 μl of medium. For cytokine production assays, 100 μl of the supernatants were added to the wells. Supernatants were collected after 24 h of incubation and assessed for human IFN-ɣ, TNF-α, and IL-2 using the ELISA MAX Deluxe set (Biolegend), following the manufacturer’s protocol. For PBMCs or T-cell proliferation assays, PBMCs or T cells were labeled with 1 μmol/L Carboxyfluorescein succinimidyl ester (CFSE) (Sigma Aldrich) and co-cultured as described above for 6 days (PBMCs) or 3 days (T cells). Cells were then stained for cell viability with LIVE/DEAD (Thermo Fisher Scientific) and for CD4 and CD8 (Biolegend). Flow cytometry analysis was performed by acquiring a total of 20,000 events.

### Cytotoxicity assays

Viral cytotoxicity assays were performed as previously described [17]. IC50 was calculated with GraphPad Prsim v6.02 (GraphPad Software Inc.) by a dose-response nonlinear regression with a variable slope.

To assess FBiTE-mediated cytotoxicity, CFSE-labeled target cells (HT1080, mCAFs (3 × 10^4^) or hCAFs (1 × 10^4^)) were cultured with 1,5 × 10^5^ T cells (E:T = 5) in 96-well plates or 48-well plates, respectively. 100 μl of mock, ICO15K or ICO15K-FBiTE supernatants were added. After 24 h of incubation, cocultures were trypsinized and stained with LIVE/DEAD® (Thermo Fisher Scientific). Cells were analyzed by flow cytometry and the percentage of CFSE^+^/LIVE and DEAD^+^ was determined.

For bystander killing assays, FAP-negative cells (HT1080 and A431) were cultured in the presence of T cells and its derivative mFAP or hFAP cells (E:T = 5) and 100 μl of supernatants were added. After 24 h, the cytotoxicity of the FAP-negative cells and mFAP or hFAP cells were determined by flow cytometry. mFAP- and hFAP-expressing cells were identified as a CFSE- hCD45- double negative cells. The percentage of CFSE^+^/LIVE and DEAD^+^ cells and CFSE^-/^hCD45^−^/LIVE and DEAD^+^ cells was determined.

FBiTE-mediated cytotoxicity of FAP-positive non-infected cells was assessed infecting A549 cells in suspension with ICO15K or ICO15K-FBiTE (MOI = 20). After 4 h, infected cells were washed thrice with PBS. 3 × 10^4^ A549-infected cells were mixed with 3 × 10^4^ CFSE-labeled target cells (1:1), T cells (E:T = 5) and supernatants (100 μl). After three days of incubation, cocultures were stained and analyzed as described above.

### Xenograft mouse models

All animal experiments were approved by the Ethics Committee for Animal Experimentation from Biomedical Research Institute of Bellvitge (IDIBELL). A549 (4 × 10^6^) or HPAC (2 × 10^6^) cells were subcutaneously injected into each flank of female, 8-week-old, NOD/*scid*/*IL2rg*^*−*^*/*^*−*^ (NSG) mice (bred in house). Once tumors reached a median volume of 120 mm^3^, mice were randomized prior to treatment.

To evaluate T-cell trafficking to the tumor, mice bearing A549 tumors were treated intratumorally with PBS, ICO15K, or ICO15K-FBiTE (1 × 10^9^ vp/tumor). Four days later, 1 × 10^7^ preactivated GFP- and CBG-luciferase-expressing T cells (LUC-T-cells) were intravenously injected to treated mice. Mice were given an intraperitoneal injection of 15 mg/mL D-luciferin potassium salt solution (Byosinth AG) and imaged daily for 7 days using IVIS Lumina XRMS Imaging System (PerkinElmer).

For antitumor efficacy studies, mice were treated intratumorally with PBS or the indicated viruses (1 × 10^9^ vp/tumor). Tumors were measured twice or thrice a week with a digital caliper and tumor volume was determined with the eq. V (mm^3^) = π/6 × W^2^ × L, where W and L are the width and the length of the tumor, respectively.

### Immunohistochemistry

To detect FAP and E1A-Adenovirus expression in tumors, immunohistochemistry (IHC) was performed using OCT-embedded sections (5 μm thick) of freshly frozen tumor tissues. Sections were fixed with 2% of PFA at room temperature and endogenous peroxidases were blocked by incubation in 3% H_2_O_2_. Next, sections were blocked for 1 h with 10% of normal goat serum diluted in 1% BSA, PBS-Tween. For FAP detection, primary antibody incubation was performed overnight at 4 °C using a biotinylated polyclonal sheep anti-human/mouse FAP antibody (5 μg/ml) or its isotype sheep IgG (R&D systems) in 5% of goat serum. For adenovirus detection, the primary antibody used was an anti-Ad2/5 E1A antibody (Santa Cruz Biotechnology) diluted 1/200 in PBS. The next day, sections were incubated with ABC-HRP kit (Vectastain) for 30 min, followed by 5 min incubation with DAKO-DAB substrate (EnVision). Slides were dehydrated using standard protocols and counterstained with haematoxylin.

### DNA/RNA quantification by qPCR

Frozen tumor samples were disrupted using a mortar and pestle under liquid nitrogen. RNA and DNA were isolated from approximately 25 mg of homogenized tissue with the DNA/RNA/protein kit (IBI Scientific). RNA samples were treated with the TURBO DNA-*free* kit (Thermo Fisher Scientific) to remove traces of genomic DNA. RNA (1 μg) was retrotranscribed with the High-Capacity cDNA Reverse Transcription kit (Thermo Fisher Scientific). Real-time analysis was performed in a LightCycler 480 Instrument II (Roche). To quantify the viral genomes and FBiTE transcripts in the tumor, 100 ng of DNA and 40 ng of cDNA in the presence of SYBR Green I Master (Roche) were used, respectively. PCR conditions were: 95 °C 10 min, 40 cycles of 95 °C 15 s, 60 °C 1 min and 72 °C 7 min. Viral genome primers were Ad18852: 5’-CTTCGATGATGCCGCAGTG-3′ and Ad19047R: 5’-ATGAACCGCAGCGTCAAACG-3′ and FBiTE primers were qBiTEF: 5’-CGGCGAGAAAGTGACAATGAC-3′ and qBiTER: 5’-TTGGTGAGGTGCCACTTTTC-3′. Standard curves for viral genomes and FBiTE were prepared by serial dilutions of known copy numbers of adenovirus plasmid and pUC57-FBiTE, respectively. To assess murine FAP expression, 25 ng of cDNA were analyzed with the TaqMan Gene Expression Assay ref. Mm01329177_m1 (Thermo Fisher Scientific). PCR conditions were: 50 °C 2 min, 95 °C 10 min, 40 cycles of 95 °C 15 s and 60 °C 1 min. A standard curve was prepared by serial dilutions of known copy numbers of a murine FAP-expressing plasmid. Human FAP-expressing plasmid was also included as negative control. In all cases, non-retrotranscribed RNA samples, in a quantity equivalent to the amount cDNA loaded in the PCR, were used for PCR to discard genomic DNA contamination.

### Statistical analysis

Statistical analyses were performed using GraphPad Prism software v6.02. All results were expressed as means ±SD or SEM, as indicated. Two-tailed unpaired Student's *t*-test was used to evaluate the statistical significance between two groups. One-way ANOVA with Tukey *post hoc* tests was used for differences between three or more groups in a single condition or time point. *P* < 0.05 was taken as the level of significance.

## Results

### Generation and characterization of an oncolytic adenovirus secreting a FAP-targeting BiTE

**Fig. 1:**
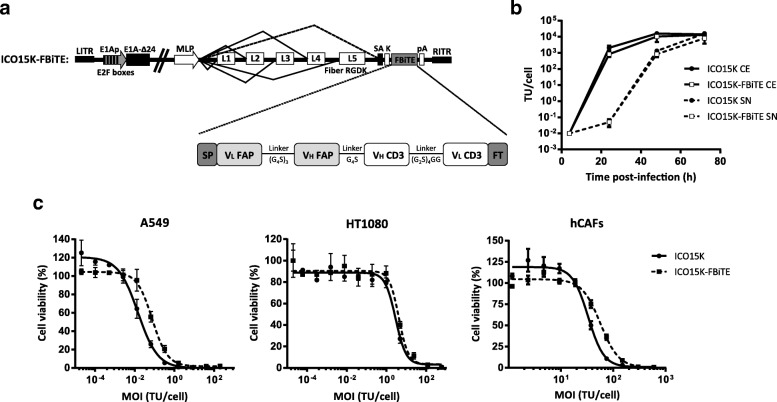
*In vitro* characterization of ICO15K-FBiTE. **a** Schematic structure representation of ICO15K-FBiTE. V_L_ and V_H_ domains of anti-mhFAP and CD3Ɛ are connected by glycine and serine flexible linkers, flanked by the light chain immunoglobulin signal peptide (SP) and the FLAG tag (FT). FBiTE is inserted after the adenovirus fiber gene under the control of the major late promoter (MLP). **b** Viral production from cell extracts (CE) and supernatants (SN) of ICO15K-FBiTE. A549 cell line was infected with ICO15K or ICO15K-FBiTE. At indicated time points, cell extracts and supernatants were harvested and titrated by an anti-hexon staining-based method. **C**. Comparative cytotoxicity profile of ICO15K-FBiTE. A549, HT1080 and hCAFs cells were infected with serial dilutions of ICO15K or ICO15K-FBiTE. Cell viability was measured at day 6 post-infection for A549 and HT1080 and at day 7 for hCAFs. Mean values ± SD are plotted in B and C (*n* = 3). TU, transducing units: number of functional viral particles capable of transducing a cell

We have recently reported the generation of an oncolytic adenovirus armed with a BiTE targeting the EGFR on tumor cells (ICO15K-cBiTE) [14]. This approach, however, does not address the presence of a tumor stroma which can impair virus spread in the tumor. In order to simultaneously target cancer cells through virus-mediated oncolysis and to re-direct immune responses towards tumor stroma fibroblasts, we engineered the genome of the oncolytic adenovirus ICO15K to express a FAP-targeting BiTE (FBiTE) (named as ICO15K-FBiTE). The FBiTE molecule was engineered by joining with flexible linkers (GS linkers) two scFv, one specific for human CD3ɛ and the other for murine and human FAP (Fig. 1a). FAP scFv sequence was specifically chosen to bind both murine and human to be able to target the murine CAFs infiltrated in xenograft tumors in the *in vivo* experiments. We have previously demonstrated that the insertion of a transgene after the fiber gene using an adenoviral splicing acceptor favors its expression in a replication-dependent manner without interfering with viral oncolysis [14, 18]. Using this strategy, the FBiTE was inserted under the control of the adenovirus major late promoter (Fig. 1a).

To evaluate whether the FBiTE insertion affected the viability and the oncolytic properties of the virus, we first compared the replication kinetics of ICO15K and ICO15K-FBiTE in A549 cells. We observed a minor, although not significant, loss in the production yields from cell extracts and supernatants of ICO15K-FBiTE compared to the parental virus (Fig. 1b). We next assessed the killing kinetics of the virus in dose-response cytotoxicity assays in three cancer cell lines (A549, HT1080 and hCAF). As shown, the FBiTE-expressing adenovirus conserved oncolytic properties despite slight increases in IC_50_ values compared to the parental virus.

**Fig. 2:**
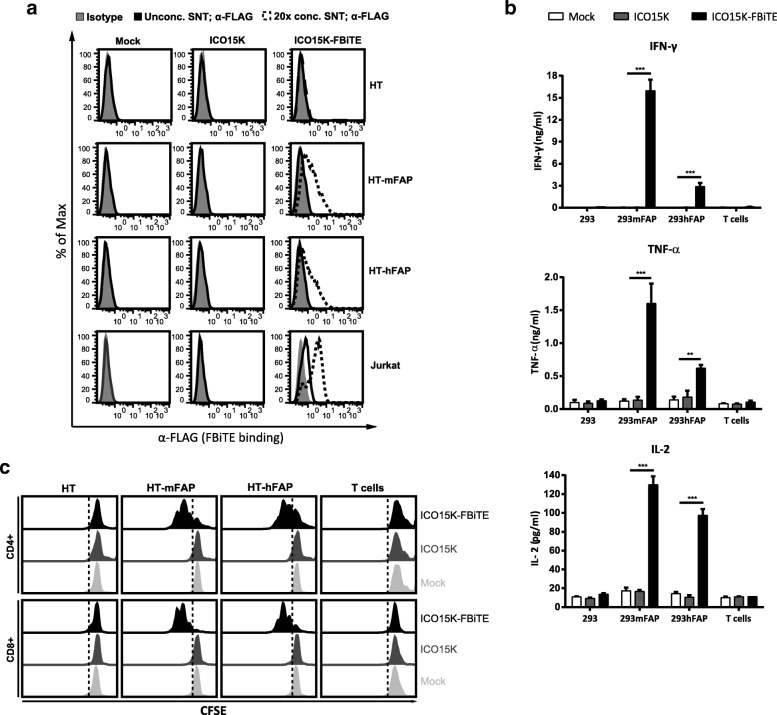
FBiTE expressed by ICO15K-FBiTE specifically binds to both target and effector cells and is able to activate T cells. **a** HT1080 (HT), HT-mFAP, HT-hFAP and Jurkat cells were incubated with mock, ICO15K, or ICO15K-FBiTE supernatants and FBiTE binding was detected by flow cytometry. **b** Average concentration values of IFN-ɣ, TNF-α and IL-2 cytokines were measured by ELISA assay using supernatants from 24 h co-cultures of HEK293 (293), 293mFAP or 293hFAP cells with T cells (E:T = 5) and indicated supernatants. **c** T-cell proliferation following co-cultures with target cells and indicated supernatants (E:T = 5). A representative result of triplicates is shown. ***, ICO15K-FBiTE significant (*P* < 0.001) versus mock or ICO15K using one-way ANOVA test with post hoc analysis

We next determined whether FBiTEs encoded by ICO15K-FBiTE were properly secreted from cancer cells upon infection, and whether they could retain their antigen-binding specificities. To this end, we performed binding assays with HT1080 cells that had been genetically modified to express either human or murine FAP. FBiTE binding was detected by flow cytometry with a fluorescently-labeled anti-FLAG antibody. FBiTE molecuels were detected in the ICO15K-FBiTE supernatants, and they bound specifically to HT-mFAP and HT-hFAP but not to the FAP-negative HT1080-parental cell line (Fig. 2a upper and middle panels). Moreover, FBiTE molecules were also able to bind to CD3-positive Jurkat cells (Fig. 2a lower panels). CD3^+^ bindings were more pronounced when supernatants were concentrated (dashed lines) but FAP^+^ bindings were only detected when concentrated.

### Supernatants from ICO15K-FBiTE-infected cells induce activation and proliferation of T cells

In order to detect the FBiTE-mediated T-cell effector functions, we evaluated both cytokine production and proliferation of T cells after co-culture with 293 cells, either expressing or not murine or human FAP, in the presence of supernatants from adenovirus-infected (ICO15K or ICO15K-FBiTE) or uninfected cells (mock). After 24 h of incubation, supernatants were collected and T-cell activation was assessed by quantifying IFN-ɣ, TNF-α, and IL-2 by ELISA (Fig. 2b). Significant cytokine release was observed in co-cultures of T cells and FAP-expressing cells and in the presence of ICO15K-FBiTE supernatants. Cytokines levels were higher in the presence of murine FAP-expressing cells compared to human FAP-expressing target cells. This in line with the affinity of the FAP5 monoclonal antibody from which the scFv in our BiTE is derived, which has been reported to be 5 nM for human FAP and 0.6 nM for mouse FAP [10]. Importantly, there was no cytokine production in the absence of FAP^+^ targets (293 control cells) or when using supernatants from a parental virus or from non-infected cells. These data demonstrate that FBiTE molecules secreted from infected cells are able to activate T cells in FAP-expression dependent manner.

To further confirm the FBiTE-mediated induction of T-cell effector functions, we evaluated T-cell proliferation after 3 days of co-culture. Both CD4^+^ and CD8^+^ T cells showed proliferation only in the co-cultures containing FAP-expressing cells and the ICO15K-FBiTE supernatants (Fig. 2c). After performing the same analysis with PBMCs instead of isolated T cells, both CD4^+^ and CD8^+^ T cells showed strong proliferation always in the co-cultures of PBMCs with FBiTE-containing supernatants, even in the absence of FAP-expressing cells (Additional file 1). These results support previous research which showed that a population of macrophages in PBMCs express FAP [19]. To avoid unspecific FBiTE activation of T cells in PBMCs, we used isolated T cells for further experiments.

### Combining viral oncolysis with FBiTE-mediated killing improves therapeutic activity *in vitro*

**Fig. 3:**
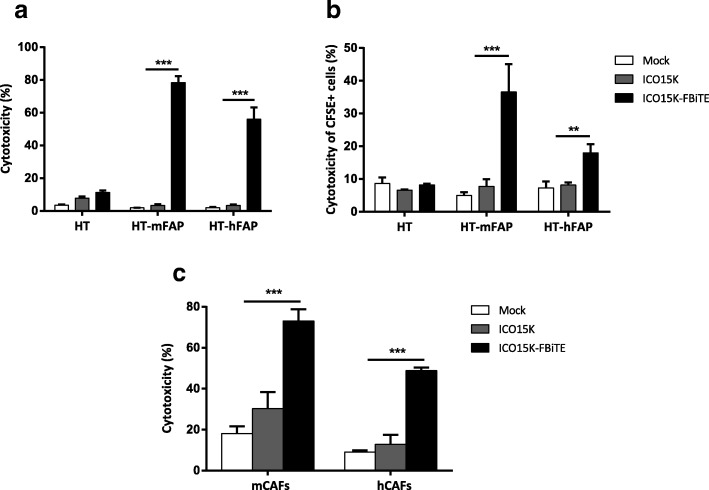
Enhanced ICO15K-FBiTE-mediated cytotoxicity of FAP-positive cells. **a** FBiTE-mediated cytotoxicity was evaluated by flow cytometry after 24 h incubation of CFSE-stained HT1080 cell lines cultured with T cells and indicated supernatants. **b** CFSE-stained target cells were co-cultured with A549-infected cells and T cells (E:T = 5). After four days of incubation, specific cytotoxicity of CFSE-stained cells was determined by flow cytometry. **c** Cytotoxicity of CFSE-stained-murine or human CAFs was evaluated. Mean values ± SD are plotted in A, B and C (*n* = 3). ***, ICO15K-FBiTE significant (*P* < 0.001) by one-way ANOVA test with *post hoc* analysis compared to mock and ICO15K

Having shown the expression of FBiTE from ICO15K-FBiTE-infected cells, we next investigated FBiTE-mediated cytotoxicity *in vitro*. We first evaluated the effect of co-culturing HT1080 and its derivative FAP-expressing cell lines with T cells and the indicated supernatants. Marked cytotoxicity of FAP-positive engineered cell lines was observed after 24 h of incubation only in the presence of ICO15K-FBiTE supernatants (Fig. 3a). A recent study demonstrated that BiTEs can also mediate a bystander tumor cell killing of nearby cells lacking the targeted antigen [20]. To evaluate this, we co-cultured CFSE stained FAP-negative cells (HT or A431) with T cells and its derivative mFAP or hFAP-positive cells, and supernatants were added. After 24 h, the cytotoxicity of the CFSE-FAP-negative cells and the mFAP or hFAP-expressing cells was determined by flow cytometry. mFAP and hFAP cells were identified as a CFSE- hCD45- double negative cells. In both cell lines we observed some cytotoxicity of FAP-negative cells (from 15 to 20%) only when co-cultured together with FAP-positive cells and ICO15K-FBiTE supernatants. This result supports an existing BiTE-dependent T cell-induced bystander lysis of FAP-negative cells proximal to FAP-positive cells (Additional file 2).

We next investigated the potential of combining viral oncolysis and FBiTE-mediated killing of FAP-positive non-infected cells. To this end, A549 cells were infected with ICO15K-FBiTE or parental ICO15K at an MOI 20. After 4 h of incubation, cells were washed and co-cultured with HT or HT-FAP-CFSE-stained cells and T cells. In this setup, A549 cells act as FBiTE producers whereas HT cells represent the target cells. The expression of OAd-infected cells specifically increased the cytotoxicity of FAP-positive target tumor cells (Fig. 3b). These results demonstrate that expression of FBiTE is compatible with viral replication and sufficient to achieve the combined oncolysis and FBiTE T-cell mediated killing of the non-infected targeted cells *in vitro*.

Although the above-mentioned experiments prove the FBiTE-mediated killing of FAP-expressing cancer cell lines, the ultimate goal of the secreted FBiTE is to target the FAP^+^ CAFs in the tumor microenvironment. To demonstrate the therapeutic potential of the FBiTE in that context, cytotoxicity experiments were performed by co-culturing murine CAFs (mCAFs) and human CAFs (hCAFs) with human T cells and the different supernatants. As shown in Fig. 3c, T-cell-mediated killing of both mCAFs and hCAFs was observed in co-cultures containing the ICO15K-FBiTE supernatant. These results not only confirm the cytotoxic potential of the secreted FBiTE, but also demonstrate that mCAFs can be targeted and killed by human T cells, a prerequisite for the use of *in vivo* xenograft models in which the stroma is from mouse origin.

### ICO15K-FBiTE increases tumor T-cell retention and accumulation in vivo

**Fig. 4:**
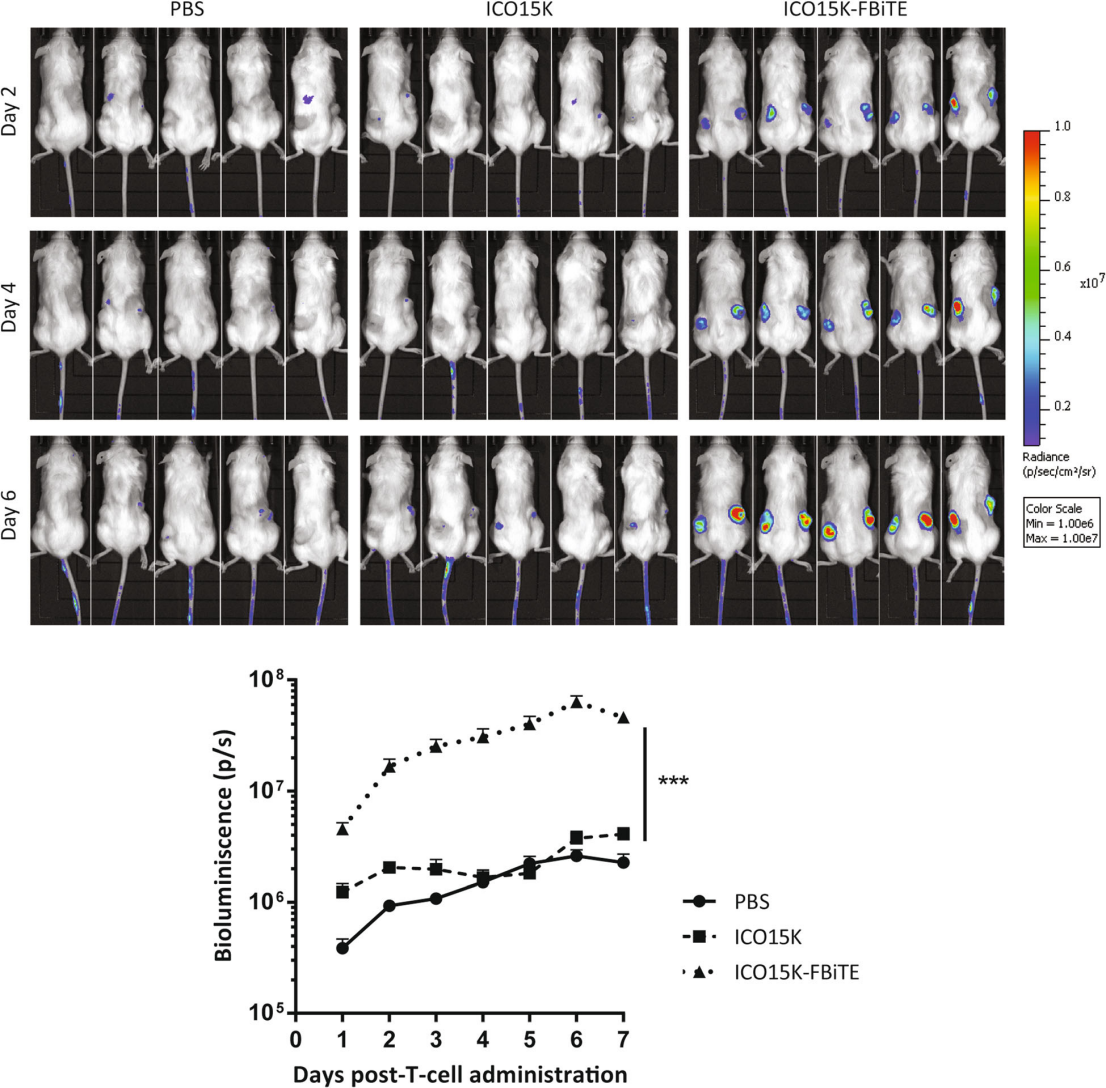
Increased T cell accumulation in ICO15K-FBiTE tumors. NSG mice bearing A549 (lung carcinoma) tumors were intratumorally treated with PBS, ICO15K, or ICO15K-FBiTE (1 × 10^9^ vp/tumor). Four days post-virus treatment, all mice received an intravenous injection of 1 × 10^7^ LUC-T-cells (64% GFP^+^). Luciferase activity was analyzed by bioluminescence imaging (IVIS) daily until day 7. Mean values ± SEM with ≥5 animals per group are shown. ***, ICO15K-FBiTE significant (*P* < 0.001) by one-way ANOVA test with *post hoc* analysis compared to PBS and ICO15K groups

In order to evaluate T-cell trafficking to ICO15K-FBiTE-treated tumors, a biodistribution imaging study was performed. Preactivated T cells were transduced with a lentiviral vector expressing GFP and the Click Beetle Green (CBG) luciferase. We obtained 64% GFP-CBG-positive cells (Additional file 3A), of which 64% were CD4^+^ and 33% were CD8^+^ (Additional file 3B). Tumors were injected with PBS, ICO15K, or ICO15K-FBiTE when reached approximately 120mm^3^, and four days post-treatment 1 × 10^7^ LUC-T cells were intravenously injected to all mice groups. Mice were imaged every day until sacrificed. ICO15K-FBiTE-treated tumors showed significant enhanced T-cell retention and accumulation from the first day post-injection, increasing daily unto reaching a peak at day 6 (Fig. 4). This result proved the feasibility of the bystander therapy in an *in vivo* scenario.

### ICO15K-FBiTE-mediated oncolysis enhances antitumor efficacy *in vivo*

**Fig. 5:**
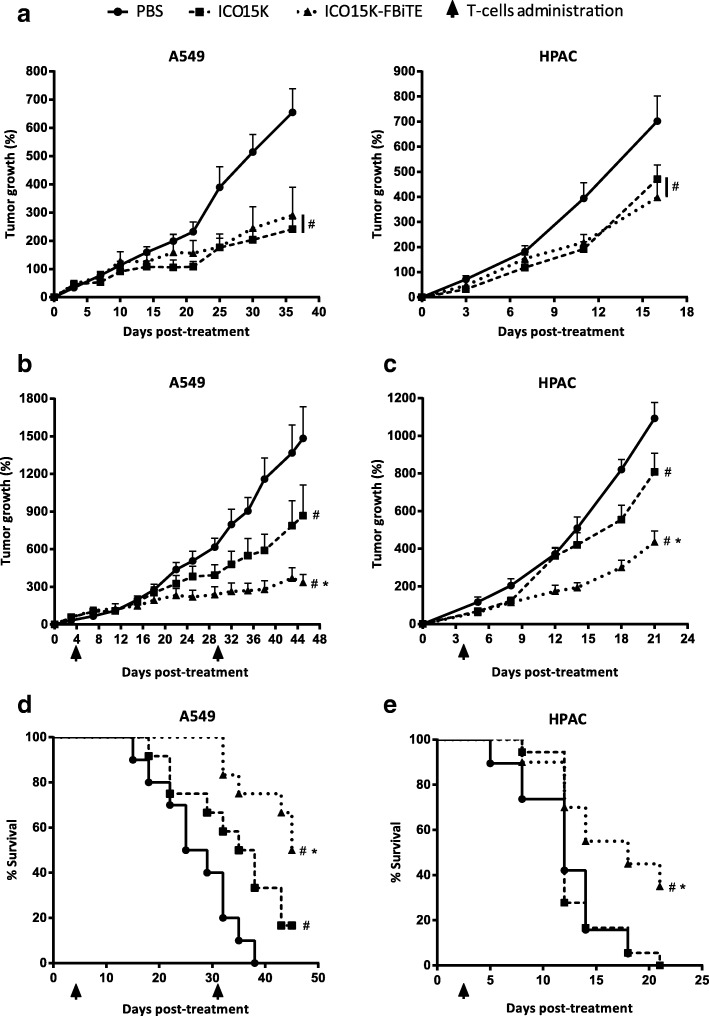
Enhanced antitumor efficacy of ICO15K-FBiTE in the presence of T cells. **a**-**e** NSG mice bearing subcutaneous A549 (lung carcinoma) or HPAC (pancreatic adenocarcinoma) tumors were intratumorally treated with PBS, ICO15K, or ICO15K-FBiTE (1 × 10^9^ vp/tumor). **a**. Antitumor activity in absence of T cells. Mean percentage of tumor growth value ± SEM with ≥12 tumors per group is plotted. **b**-**e** Antitumor efficacy in the presence of T cells. Four days after virus treatment, animals were treated once (HPAC) or twice (A549) with 1 × 10^7^ preactivated T cells. The mean tumor growth ± SEM of ≥12 tumors per group is shown. **d**, **e** Kaplan-Meier survival curves of the experiments described in **b** and **c**. *, significant (*P* < 0.05) by one-way ANOVA test with *post ho*c analysis compared to ICO15K group. #, significant (*P* < 0.05) by one-way ANOVA test with *post hoc* analysis compared to PBS group

We next assessed whether the accumulation of FAP-targeted-T cells observed in ICO15K-FBiTE-treated mice could improve the antitumor efficacy in A549 (human lung cancer) and HPAC (human pancreatic) tumor models. It has previously shown that these tumor models generate FAP^+^ stroma once implanted subcutaneosly in NSG mice [5, 7]. Tumor-bearing mice were randomized into treatment groups and treated with a single intratumoral administration of PBS, ICO15K or ICO15K-FBiTE (1 × 10^9^ vp/tumor) when the tumor volume reached a mean of 120mm^3^. We first evaluated the antitumor activity of our viruses in both tumor models in the absence of T cells (Fig. 5a). The treatment with either the FBiTE-armed or parental viruses induced a similar significant level of efficacy compared to the PBS group (Fig. 5a). Then we assessed the antitumor activity only in presence of T cells (Fig. 5b-e), therefore it should not be directly compared to Fig. 5a. Four days post-virus-treatment, 1 × 10^7^ preactivated T cells were injected intravenously once (HPAC) or twice (A549) to all mice-treated groups. Based on our pilot studies which classified HPAC tumors as fast growing, only a single dose of T cells was performed. We observed no significant weight loss in these experiments (Additional file 4). In the A549 model, a model that grows much more slowly than HPAC, there were no differences in tumor growth among the different groups up to day 15 (Fig. 5b, Additional file 5a). After this day, the mean tumor growth among treatment groups were statistically different. In contrast, in the fast-growing HPAC model, significant differences started earlier, from day 9 (Fig. 5d, Additional file 5B) but tumor growth was more difficult to control. In both tumor models, tumors growth of tumors treated with the FBiTE-expressing adenovirus were significantly smaller when compared with the tumors treated with PBS or with the control virus. This treament also improved significantly the survival (Fig. 5c and e), providing evidence for the therapeutic benefit of arming an oncolytic adenovirus with the FBiTE.

### ICO15K-FBiTE improves the antitumor activity by depletion of FAP

**Fig. 6:**
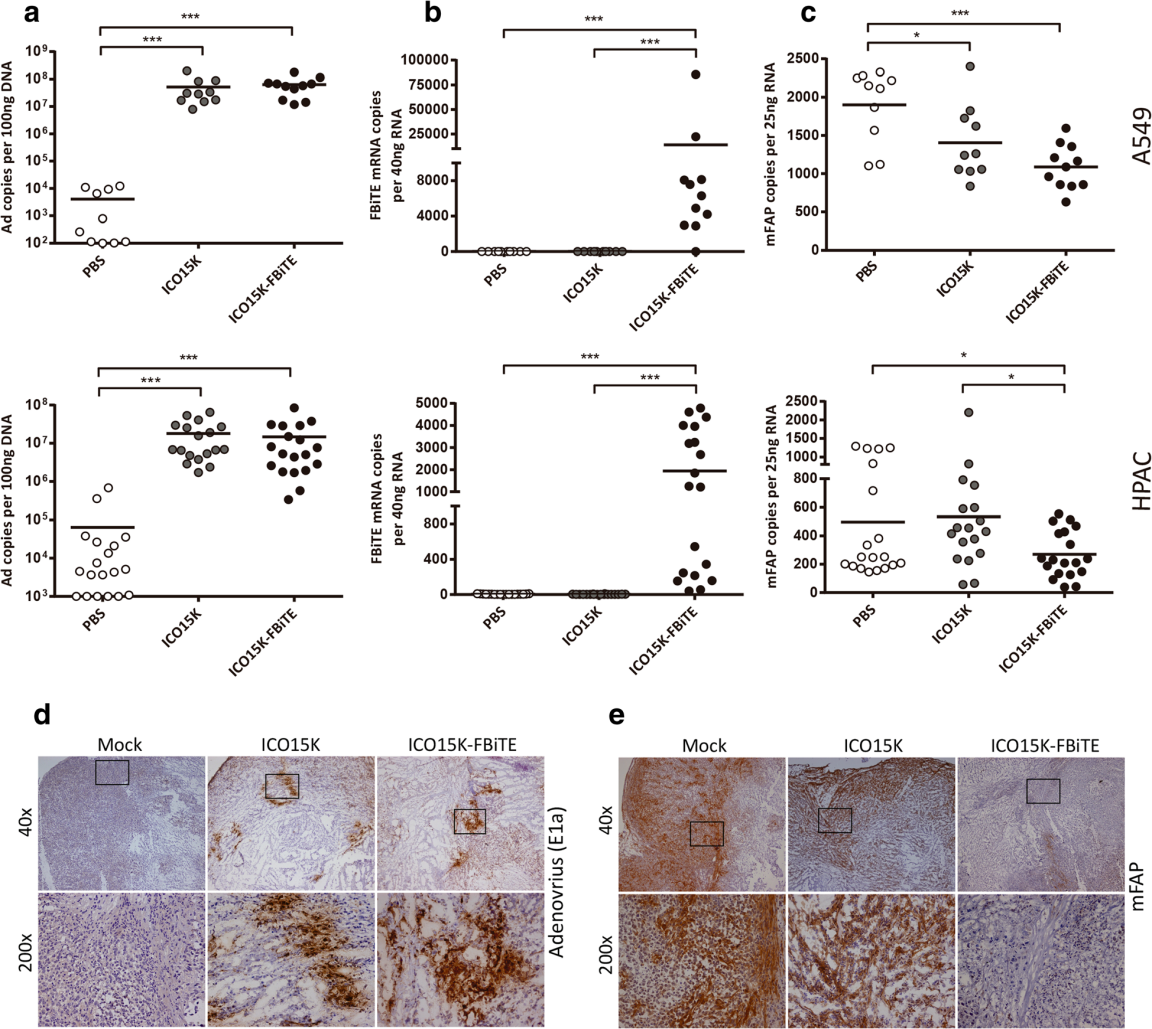
Depletion of tumor stroma by ICO15K-FBiTE. A549 and HPAC tumors from the antitumor efficacy studies in presence of T cells were harvested at endpoint of the experiments. **a-c** Piece of tumor samples were mechanically homogenized, total DNA and RNA were extracted and quantification by real-time PCR-based method was performed to evaluate **a** the virus persistence, **b** FBiTE expression and **c** levels of FAP expression in both tumor models. **d, e** Immunohistochemical stainings of A549 tumors were also performed to assess **d** presence of virus by staining the early viral protein E1a and **e** FAP expression by an anti-FAP antibody. *, significant (*P* < 0.05) by two-tailed unpaired Student’s *t*-test. ***, significant (*P* < 0.001) by two-tailed unpaired Student’s *t*-test

We analysed tumor samples from the efficacy studies described above to demonstrate that the observed improved antitumor activity was associated to the elimination of CAFs by T cells retargeted with the FBiTE expressed from the oncolytic adenovirus. We first quantified the viral genomes and the FBiTE copy numbers by real-time PCR. As expected, we observed high amounts of viral genomes only in virus-treated tumors compared to PBS-treated tumors (Fig. 6a), indicating that both viruses are able to infect and replicate in both tumor models. This result is further supported by similar findings when the presence of virus was evaluated by an anti-E1A immunohistochemistry (Fig. 6d). As expected, we could detect FBiTE expression only in ICO15K-FBiTE-treated tumors (Fig. 6b). These data confirm that viruses are present in the tumor and that the FBiTE is locally expressed in vivo upon ICO15K-FBiTE infection.

Having shown the in vivo persistence of both viruses as well as the FBiTE expression by the modified virus, we next sought to demonstrate the hypothesis that the enhanced antitumor effect was associated to depletion of FAP. FAP expression was first quantified by real-time PCR. As shown in Fig. 6c, the expression of FAP was reduced in both tumor models, in ICO15K-FBiTE-treated tumors compared with the PBS and the control virus. Consistent with this mRNA quantification data, the amount of FAP protein detected by staining the A549 tumors was also lower (Fig. 6e). Altogether, these results indicate that the T cells retargeted by the FBiTE are responsible for killing the FAP-positive murine CAFs in the tumor mass.

## Discussion

Oncolytic adenoviruses (OAds) represent promising therapeutic agents that promote antitumor effects through a dual mechanism: selective tumor cell killing and the induction of antitumor immunity. However, several clinical trials with OAds generated promising albeit modest results [21]. Novel strategies are therefore needed to overcome the obstacles that prevent successful application of OAds, such as eliminating the tumor stroma that prevents efficient virus spread and preventing the immunodominance of the adenoviral epitopes that promotes fast virus clearance [22, 23].

In the present work, we aimed at addressing these limitations by arming an oncolytic adenovirus with an anti-FAP Bispecific T-cell Engager (FBiTE). In contrast to other therapies, one of the most important advantages of using BiTEs is its MHC-I-independent mode of action [24, 25]. BiTEs force T cells and tumor cells to come in close contact, forming an immunological synapse that shows all the hallmarks of a synapse formed by T cell receptor-MHC class I-peptide induced synapses. Therefore, encoding a BiTE specific against FAP would ideally re-direct lymphocytes to become cytotoxic against the tumor stroma, improving virus spread in the tumor microenvironment. In this study, we have demonstrated that once the FBiTE is expressed and secreted from infected cells, it can successfully activate both CD4 and CD8 T cells. This activation leads to T-cell-mediated cytotoxicity of the FAP-expressing cells in vitro and in vivo and T-cell-induced bystander cell lysis of FAP-negative cells. Moreover, we have also demonstrated that the depletion of FAP-positive stromal cells from the tumor mass enhances the overall antitumor efficacy without increasing the toxicity. This data confirms previous results suggesting that targeting FAP enhances antitumor efficacy and might be therefore a promising approach for clinical benefits [3–10].

The use of OVs to achieve the local stimulation of the immune system against the tumor is a hot research field, leading to strong and durable responses [26]. OVs-infected cells create an inflammatory site with the consequent release of cytokines that activate the immune system, reverting the immune-suppressive tumor environment from a “cold” to a “hot” or lymphocyte-infiltrated tumor. However, the main side effect of the host immune system is the efficient clearance of the virus counteracting the oncolytic effect of the treatment. One of the advantages of arming an oncolytic virus with a BiTE is to balance the antiviral to antitumor immunity by its ability to re-direct the infiltrated antiviral lymphocytes to kill cells that express the protein targeted by the BiTE.

It is worth highlighting that arming oncolytic viruses with BiTEs represents a combined anti-cancer therapy. Our results show that simultaneously targeting the cancer cells with the oncolytic adenovirus and the tumor stroma with the FBiTE enhances the overall antitumor efficacy. Other BiTEs encoded by oncolytic viruses have already been published. The first one was the Ephrin A2-BiTE-armed oncolytic vaccinia virus, which induced PBMCs activation and tumor cell cytotoxicity *in vitro* and *in vivo* [27]*.* In line with that study, similar results have been described with different OVs armed with BiTEs [6, 14, 28]. However, all those studies exploited BiTEs targeting tumor-specific antigens [6, 14, 27, 28]. Thus, the secreted BiTEs can target both infected and uninfected cells, thereby reducing the virus-driven BiTE production and availability in the tumor microenvironment. To overcome this limitation, the FBiTE was designed to be expressed by the infected cancer cells and to target stromal cells, thereby avoiding the depletion of BiTE-expressing cancer cells and promoting continous BiTE production dependent on viral oncolysis. In this regard, a recent report described the benefits of targeting the tumor stroma with a FAP-targeting BiTE-armed vaccinia virus in an immunocompetent mouse model of cancer [3].

One of the major concerns when targeting non-specific tumor antigens is the potential toxicity. Despite the controversies of toxicity effects related with immune targeting, fatal adverse effects have already been reported in several studies [29]. In this regard, successful growth inhibition without signs of toxicity by FAP-targeted CARs T-cells has been reported [4, 5, 8]. In contrast, Tran et al reported that FAP-targeting with FAP5-CAR-transduced T cells led to cachexia and lethal bone toxicities due to FAP expression by multipotent bone marrow stem cells (BMSCs) [7]. Other studies have shown that FAP is expressed by some normal tissues and macrophages [19, 30, 31]. In agreement with this, we found activation and proliferation of T cells when PBMCs were co-cultured with FBiTE-containing supernatants (Additional file 1). Importantly, ICO15K-FBiTE treatment did not result in any significant off-target toxicity in mice, based on body weight and general animal behavior (Additional file 4). This discrepancy can be explained by the mode of action of our OV. FBiTE expression depends on the replication of the OAd in cancer cells within the tumor microenvironment, in contrast to CART cells, which circulate freely through the body. Thus, the strategy of arming oncolytic viruses with a FAP-targeting BiTE allows the continuous expression of BiTE directly in the tumor, preventing the targeting of healthy cells by the BiTE and in turn avoiding possible adverse effects. In addition, we showed that a single dose of oncolytic adenovirus is enough to obtain a continuous expression of BiTE by infected cells, avoiding the needed of repeated systemic infusion due to short half-life of BiTEs in serum [32].

Despite the notable improvement of antitumor efficacy obtained with ICO15K-FBiTE, no complete responses were observed. These findings may be somewhat limited by the lack of adequate tumor models used. Using immunocompetent models in order to explore the impact of infiltrating T cells in the tumor after virus injection would represent a more realistic scenario. However, the species-specific nature of the adenovirus infection and replication restricts the appropriate evaluation in immunocompetent mouse models. The limited and transient presence of adoptively transferred lymphocytes in our model could explain the decrease but incomplete elimination of FAP^+^ cells in treated-tumors. Another reason that could explain the incomplete tumor rejection could be related to the insufficient activation of T cells. A recent report has demonstrated the importance of co-stimulation during BiTE-engagement in order to obtain improved antitumor efficacy [33]. This study highlights the need of developing improved BiTE constructs in order to avoid T cell exhaustion due to chronic antigen stimulation. In this line, combining this therapy with other immuno- or chemotherapies may also represent significant advantages. For example, we have recently demonstrated that combining BiTE-armed OV with CART cells improve CART-cell activation and proliferation in vitro and in vivo, thereby enhancing T-cell-mediated cytotoxicity [34]*.* We and others have also shown an increase in the expression of T-cell inhibitory receptors after immune-based therapies, likely limiting the antitumor activity [34, 35]. For example, Ribas et al reported the strong enhanced immune recognition of cancer when combined talimogene laherparepvec oncolytic virus with an anti-PD1 antibody [26]. These studies support the rationale to combine our BiTE-expressing virus with different immune checkpoint inhibitors. On the other hand, Fang et al reported the benefits of combining FAP-targeted therapies with chemotherapies [11]. Such results suggest that destroying the stroma not only improves virus spread but also may allow chemotherapy drugs to better penetrate into tumor. It is therefore likely that the successful application of FAP-targeted by BiTE-armed oncolytic adenovirus in cancer patients will require the development of an optimized therapeutic approach.

## Conclusion

This study establishes ICO15K-FBiTE as an effective strategy for targeting both cancer cells and FAP-positive stromal cells, killing through combined viral oncolysis and intratumoral expression of an anti-FAP BiTE. This approach offers opportunities for cancer therapy with no evidence of toxicity and further encourages the transition into clinical applications. Future studies should be directed towards optimization of both oncolytic adenovirus and BiTE designs and to explore the effectiveness of FAP-targeting BiTE-armed oncolytic adenovirus in combination with other therapeutic modalities, such as chemotherapy or other immunotherapies.

## Additional files

Additional file 1: FBiTEs molecules expressed from ICO15K-FBiTE-infected cells induce T-cells proliferation when co-cultured with PBMCs. 293, 293mFAP and 293hFAP were co-cultured with CFSE-labeled PBMCs and indicated supernatants. Six days after co-culture, the CFSE content in CD4^+^ and CD8^+^ T-cells was determined by flow cytometry. A representative result of triplicates is shown. (DOCX 13487 kb)

Additional file 2: FBiTE-mediated bystander tumor cell killing. A, B. CFSE-stained HT cells (A) or A431 cells (B) were culture in the presence of T cells and its derivative mFAP- or hFAP cells and the indicated supernatants (mock, ICO15K or ICO15K-FBiTE) were added. After 24 h, cytotoxicity of HT cells (A) or A431 cells (B) and its mFAP- or hFAP-derivative cells were evaluated by flow cytometry. Mean values ± SD are plotted in A, B (*n* = 3). ***, significant (*P* < 0.001) by one-way ANOVA test with post hoc analysis compared to mock and ICO15K. **, significant (*P* < 0.01) by one-way ANOVA test with post hoc analysis compared to mock and ICO15K. (DOCX 168 kb)

Additional file 3: Characterization of GFP- and CBG Luciferase-expressing T cells. A. Flow cytometry analysis of GFP expression of preactivated T-cells that had been transduced with a lentiviral vector encoding GFP and the click beetle green (CBG) luciferase. B. Percentages of CD4 and CD8 LUC-T-cells populations determined by flow cytometry. (DOCX 231 kb)

Additional file 4: Body weight variation in A549 xenograft antitumoral efficacy assay. Animal body weight was monitored weekly after intratumoral injection of PBS, ICO15K or ICO15K-FBiTE (2 × 10^9^ vp). Mean values ± SEM are plotted (*n* = 6–7). (DOCX 140 kb)

Additional file 5: Antitumor activity of ICO15K-FBiTE. NSG mice bearing subcutaneous xenografts of A549 or HPAC tumors were injected intratumorally with PBS or 2 × 10^9^ viral particles of ICO15K or ICO15K-FBiTE. The mean tumor volume ± SEM of ≥12 tumors per group is shown. *, significant *(P* < 0.05*)* by one-way ANOVA test with post hoc analysis compared to ICO15K group. #, significant *(P* < 0.05*)* by one-way ANOVA test with post hoc analysis compared to PBS group. (DOCX 195 kb)

## Abbreviations

ALL: Acute lymphoid leukemia
ATCC: American Type Culture Collection
BiTE: Bispecific T-cell engager
BST: Blood and Tissue Bank
CAF: Cancer-associated fibroblast
CBG: Click beetle green luciferase
CFSE: Carboxyfluorescein succinimidyl ester
ECM: Extracellular matrix
FAP: Fibroblast activation protein-α
FBiTE: FAP-targeting bispecific T-cell engager
GM-CSF: granulocyte macrophage colony stimulating factor
hCAFS: human CAFs
HSV: Herpes simplex virus
ICO15K-cBiTE: EGFR-BiTE-armed oncolytic adenovirus
ICO15K-FBiTE: FAP-BiTE-armed oncolytic adenovirus
IDIBELL: Biomedical Research Institute of Bellvitge
IHC: immunohistochemistry
LUC-T-cells: GFP- and CBG-luciferase-expressing T cells
mCAFs: murine CAFs
MOI: Multiplicity of infection
NSG: NOD/*scid*/*IL2rg*^*−*^*/*^*−*^
OAd: Oncolytic adenovirus
OV: Oncolytic virus
PBMCs: Peripheral blood mononuclear cells
ScFv: Single-chain variable fragment
TAA: tumor-associated antigen
