# Supplementary material for: Targeting the tumor stroma with an oncolytic adenovirus secreting a fibroblast activation protein-targeted bispecific T-cell engager
Source: J Immunother Cancer. 2019 Jan 25;7:19. doi: 10.1186/s40425-019-0505-4 (PMC6347837; doi:10.1186/s40425-019-0505-4)
Supplement: Supplementary file 1 — FBiTEs molecules expressed from ICO15K-FBiTE-infected cells induce T-cells proliferation when co-cultured with PBMCs. 293, 293mFAP and 293hFAP were co-cultured with CFSE-labeled PBMCs and indicated supernatants. Six days after co-culture, the CFSE content in CD4+ and CD8+ T-cells was determined by flow cytometry. A representative result of triplicates is shown. (DOCX 13487 kb) [file 40425_2019_505_MOESM1_ESM.docx]

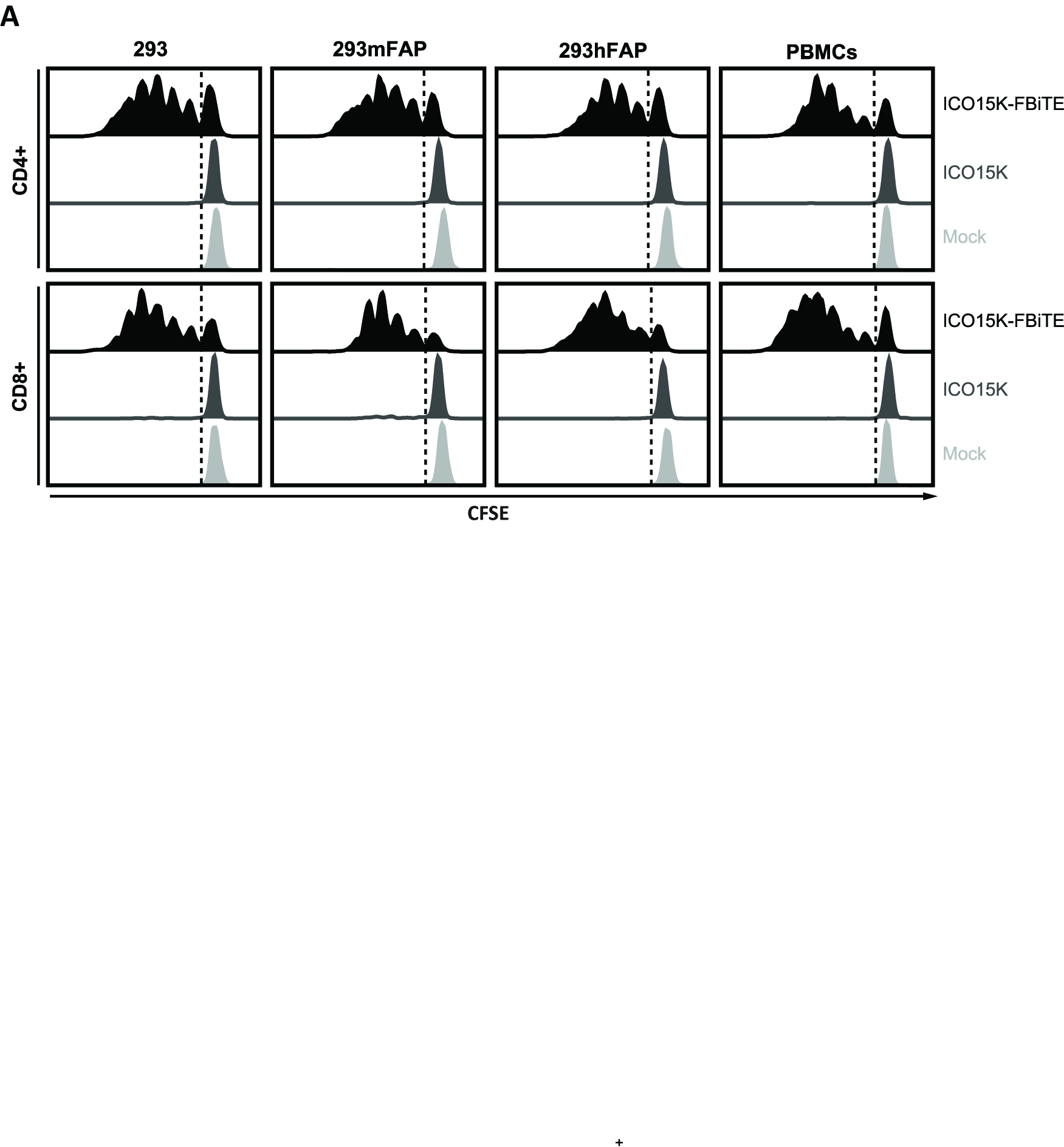


**Additional file 1.** FBiTEs molecules expressed from ICO15K-FBiTE-infected cells induce T-cells proliferation when co-cultured with PBMCs. 293, 293mFAP and 293hFAP were co-cultured with CFSE-labeled PBMCs and indicated supernatants. Six days after co-culture, the CFSE content in CD4^+^ and CD8^+^ T-cells was determined by flow cytometry. A representative result of triplicates is shown.
