# Supplementary material for: Targeting the tumor stroma with an oncolytic adenovirus secreting a fibroblast activation protein-targeted bispecific T-cell engager
Source: J Immunother Cancer. 2019 Jan 25;7:19. doi: 10.1186/s40425-019-0505-4 (PMC6347837; doi:10.1186/s40425-019-0505-4)
Supplement: Supplementary file 2 — FBiTE-mediated bystander tumor cell killing. A, B. CFSE-stained HT cells (A) or A431 cells (B) were culture in the presence of T cells and its derivative mFAP- or hFAP cells and the indicated supernatants (mock, ICO15K or ICO15K-FBiTE) were added. After 24 h, cytotoxicity of HT cells (A) or A431 cells (B) and its mFAP- or hFAP-derivative cells were evaluated by flow cytometry. Mean values ± SD are plotted in A, B (n = 3). ***, significant (P < 0.001) by one-way ANOVA test with post hoc analysis compared to mock and ICO15K. **, significant (P < 0.01) by one-way ANOVA test with post hoc analysis compared to mock and ICO15K. (DOCX 168 kb) [file 40425_2019_505_MOESM2_ESM.docx]

**
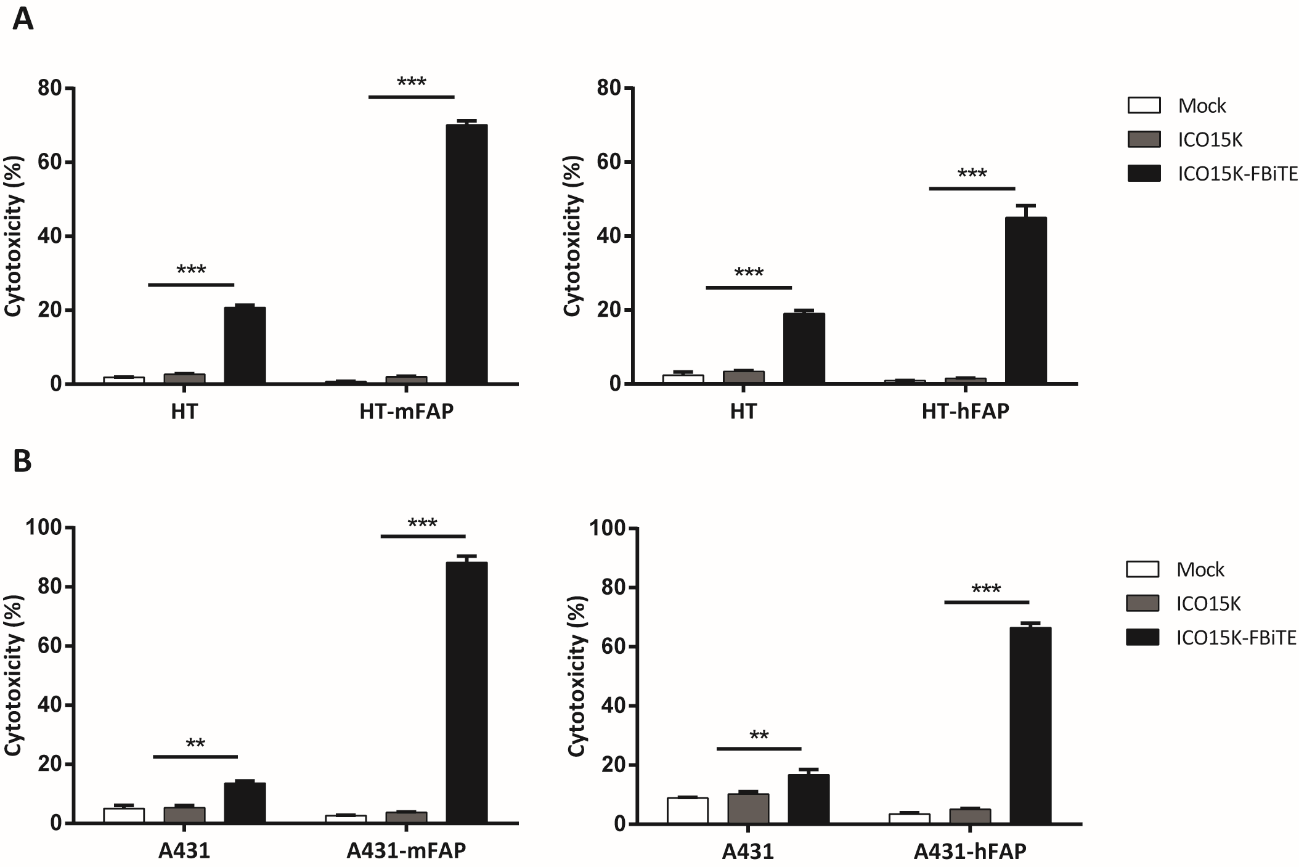
**

**Additional file 2.** FBiTE-mediated bystander tumor cell killing. **A, B.** CFSE-stained HT cells (A) or A431 cells (B) were culture in the presence of T cells and its derivative mFAP- or hFAP cells and the indicated supernatants (mock, ICO15K or ICO15K-FBiTE) were added. After 24h, cytotoxicity of HT cells (A) or A431 cells (B) and its mFAP- or hFAP-derivative cells were evaluated by flow cytometry. Mean values ± SD are plotted in A, B (n=3). ***, significant (*P* < 0.001) by one-way ANOVA test with *post hoc* analysis compared to mock and ICO15K. **, significant (*P* < 0.01) by one-way ANOVA test with *post hoc* analysis compared to mock and ICO15K.
