# Supplementary material for: Targeting the tumor stroma with an oncolytic adenovirus secreting a fibroblast activation protein-targeted bispecific T-cell engager
Source: J Immunother Cancer. 2019 Jan 25;7:19. doi: 10.1186/s40425-019-0505-4 (PMC6347837; doi:10.1186/s40425-019-0505-4)
Supplement: Supplementary file 3 — Characterization of GFP- and CBG Luciferase-expressing T cells. A. Flow cytometry analysis of GFP expression of preactivated T-cells that had been transduced with a lentiviral vector encoding GFP and the click beetle green (CBG) luciferase. B. Percentages of CD4 and CD8 LUC-T-cells populations determined by flow cytometry. (DOCX 231 kb) [file 40425_2019_505_MOESM3_ESM.docx]

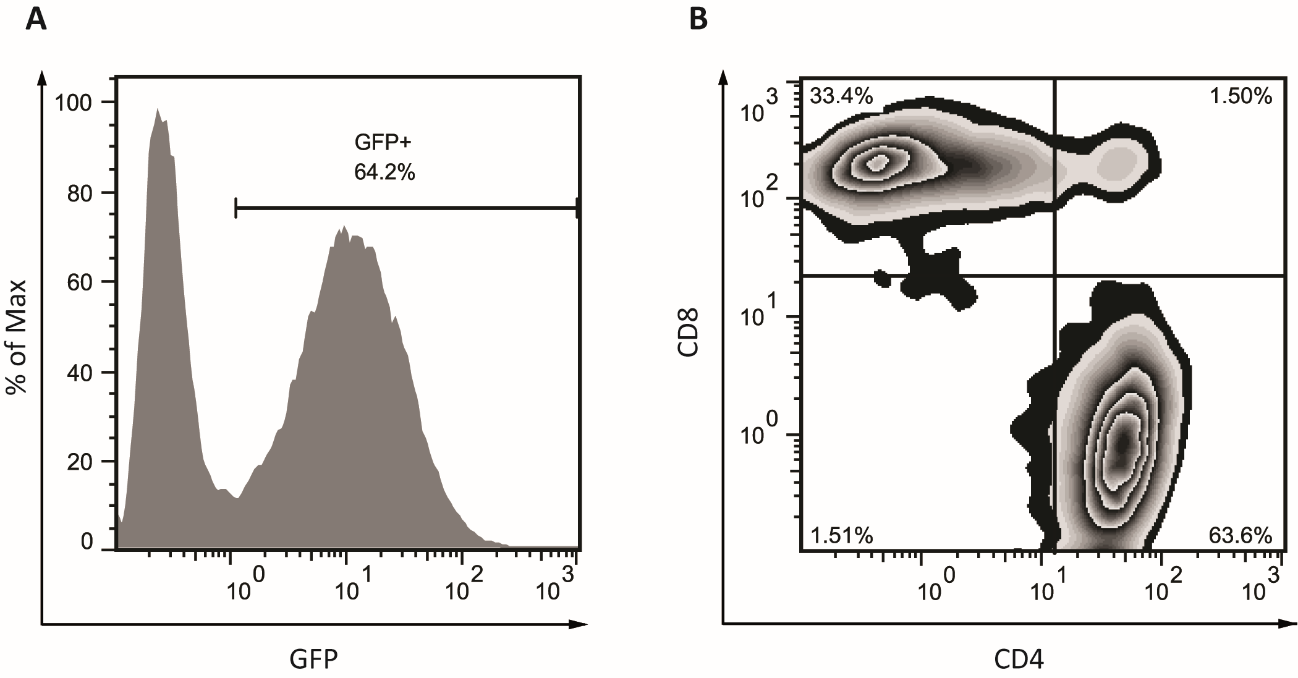


**Additional file 3.** Characterization of GFP- and CBG Luciferase-expressing T cells. **A**. Flow cytometry analysis of GFP expression of preactivated T-cells that had been transduced with a lentiviral vector encoding GFP and the click beetle green (CBG) luciferase. **B**. Percentages of CD4 and CD8 LUC-T-cells populations determined by flow cytometry.
