# Supplementary material for: Targeting the tumor stroma with an oncolytic adenovirus secreting a fibroblast activation protein-targeted bispecific T-cell engager
Source: J Immunother Cancer. 2019 Jan 25;7:19. doi: 10.1186/s40425-019-0505-4 (PMC6347837; doi:10.1186/s40425-019-0505-4)
Supplement: Supplementary file 4 — Body weight variation in A549 xenograft antitumoral efficacy assay. Animal body weight was monitored weekly after intratumoral injection of PBS, ICO15K or ICO15K-FBiTE (2 × 10^9^ vp). Mean values ± SEM are plotted (n = 6–7). (DOCX 140 kb) [file 40425_2019_505_MOESM4_ESM.docx]

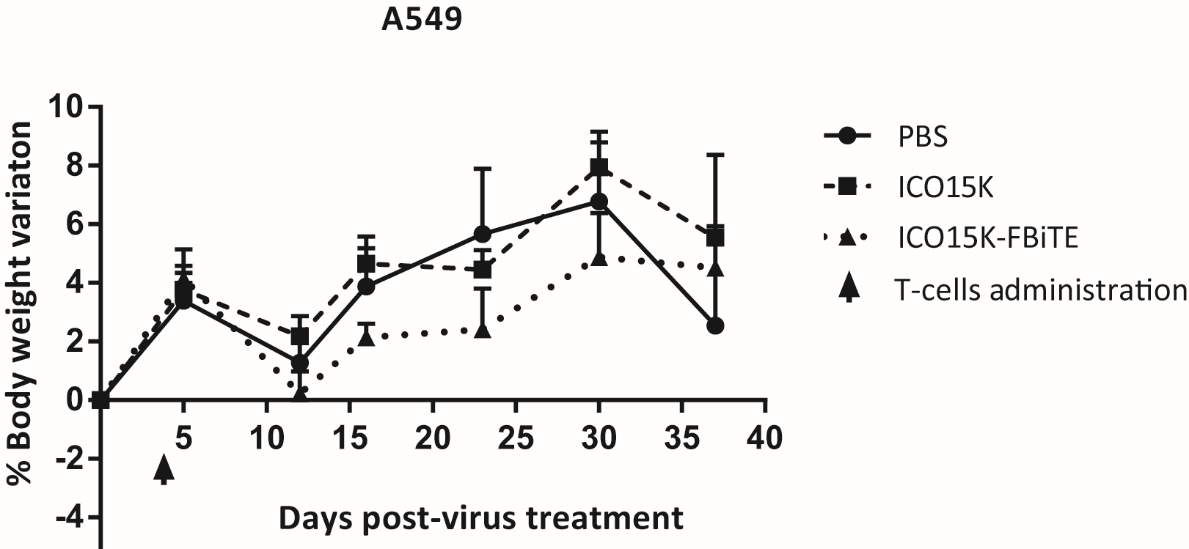


**Additional file 4.** Body weight variation in A549 xenograft antitumoral efficacy assay. Animal body weight was monitored weekly after intratumoral injection of PBS, ICO15K or ICO15K-FBiTE (2x10^9^ vp). Mean values ± SEM are plotted (n=6-7).
