# Supplementary material for: Targeting the tumor stroma with an oncolytic adenovirus secreting a fibroblast activation protein-targeted bispecific T-cell engager
Source: J Immunother Cancer. 2019 Jan 25;7:19. doi: 10.1186/s40425-019-0505-4 (PMC6347837; doi:10.1186/s40425-019-0505-4)
Supplement: Supplementary file 5 — Antitumor activity of ICO15K-FBiTE. NSG mice bearing subcutaneous xenografts of A549 or HPAC tumors were injected intratumorally with PBS or 2 × 10^9^ viral particles of ICO15K or ICO15K-FBiTE. The mean tumor volume ± SEM of ≥12 tumors per group is shown. *, significant (P < 0.05) by one-way ANOVA test with post hoc analysis compared to ICO15K group. #, significant (P < 0.05) by one-way ANOVA test with post hoc analysis compared to PBS group. (DOCX 195 kb) [file 40425_2019_505_MOESM5_ESM.docx]

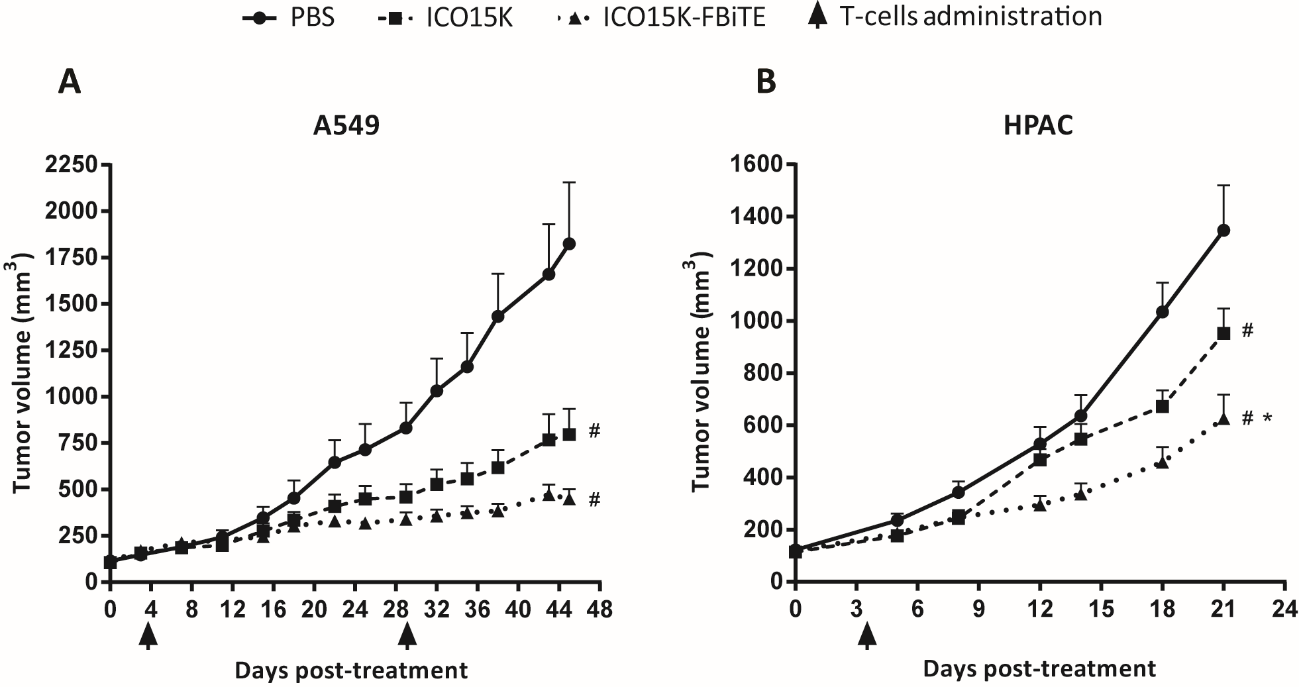


**Additional file 5.** Antitumor activity of ICO15K-FBiTE. NSG mice bearing subcutaneous xenografts of A549 or HPAC tumors were injected intratumorally with PBS or 2x10^9^ viral particles of ICO15K or ICO15K-FBiTE. The mean tumor volume ± SEM of ≥ 12 tumors per group is shown. *, significant *(P* < 0.05*)* by one-way ANOVA test with *post hoc* analysis compared to ICO15K group. #, significant *(P* < 0.05*)* by one-way ANOVA test with *post hoc* analysis compared to PBS group.
